# Notices, &c.

**Published:** 1859-02

**Authors:** 


### NOTICES, &c.

*Phonography* in five parts. By ANDREW J. GRAHAM, conductor of the Phonetic Academy, New York; author of "*Brief Longhand.*" A book on this subject, able, systematic, comprehensive, and clear, has long been a want, which the author has now fully met. Sent post-paid for $1 25.

"*Seven Miles Around Jerusalem;*" a map, 21 by 24 inches, in book form, for $1. By JAMES CHALLEN & SONS, Philadelphia. A most valuable aid to every Bible student in localizing some of the most interesting incidents of New Testament history. The same house furnishes for one dollar each the most beautiful and finished steel engravings of the leading men of the "Christian" denomination, beginning with Alexander Campbell, who, like Saul of old, stands a head and shoulders above them all in learning, courage, and mental power.

*Sargent's School Monthly*, $1 a year, Boston, we heartily commend to every growing family in the land. It is instructive to all.

"*Blackwood*" and the four reviews—Edinburgh, London Quarterly, Westminster, and North British, $10 a year, LEONARD SCOTT & Co.—affords a large amount of valuable reading to all educated men.

*Educational.*—We have never yet met with a man who could inform us where, in the city of New York, a young girl could get a thorough education in any one thing short of having a special teacher. Too many of the female boarding schools and "Institutes" are schools for sham, and smatter, and show—skimming in every thing, thorough in nothing; the theatres, where meet the snobbery of recent wealth and the pretentiousness of those once rich, but have lost every thing but their pride, making a repulsive alliance for mutual advantage. But this the really rich and elevated would be very willing to submit to, if their daughters could, in these institutions, become thorough in anything, from orthography upwards. The subject of the education of our children is not understood by over one in a thousand; and until it is, it would be better, at least in cities, for each church to assume the exclusive control over its own young, as to their literary and doctrinal instruction, aiming to have both radical and thorough as far as they went; and even although that did not go beyond first principles, it would be greatly preferable to the present system, and we hope that earnest Christian people will give it their serious consideration.

*Repudiation.*—A writer in the Home Journal states, that an eminent physician in Virginia intimated to him that the "half-educated and slenderly supported country doctors find it to their interest to prolong disease." How a man represented to be an "excellent conversationist," a "philosopher," and "scientific observer," and about retiring from the successful practice of medicine, should make such a charge against "country physicians," who perform more hazardous personal labor, without any other reward than a love of humanity and a desire of maintaining professional honor, than any other class of men, without exception, we cannot conjecture. Such a man is neither a "Virginian" nor a "gentleman;" and, if he is an educated physician, he is there by mistake, and is unworthy of professional recognition.

